# Inheritance of an Epigenetic Mark: The CpG DNA Methyltransferase 1 Is Required for De Novo Establishment of a Complex Pattern of Non-CpG Methylation

**DOI:** 10.1371/journal.pone.0001136

**Published:** 2007-11-07

**Authors:** Valérie Grandjean, Ruken Yaman, François Cuzin, Minoo Rassoulzadegan

**Affiliations:** 1 Inserm, U636, Nice, France; 2 Laboratoire de Génétique du Développement Normal et Pathologique, Université de Nice-Sophia Antipolis, Nice, France; 3 Equipe Labellisée Ligue Nationale Contre le Cancer, Nice, France; National Institute on Aging, United States of America

## Abstract

Site-specific methylation of cytosines is a key epigenetic mark of vertebrate DNA. While a majority of the methylated residues are in the symmetrical (meC)pG:Gp(meC) configuration, a smaller, but significant fraction is found in the CpA, CpT and CpC asymmetric (non-CpG) dinucleotides. CpG methylation is reproducibly maintained by the activity of the DNA methyltransferase 1 (Dnmt1) on the newly replicated hemimethylated substrates (meC)pG:GpC. On the other hand, establishment and hereditary maintenance of non-CpG methylation patterns have not been analyzed in detail. We previously reported the occurrence of site- and allele-specific methylation at both CpG and non-CpG sites. Here we characterize a hereditary complex of non-CpG methylation, with the transgenerational maintenance of three distinct profiles in a constant ratio, associated with extensive CpG methylation. These observations raised the question of the signal leading to the maintenance of the pattern of asymmetric methylation. The complete non-CpG pattern was reinstated at each generation in spite of the fact that the majority of the sperm genomes contained either none or only one methylated non-CpG site. This observation led us to the hypothesis that the stable CpG patterns might act as blueprints for the maintenance of non-CpG DNA methylation. As predicted, non-CpG DNA methylation profiles were abrogated in a mutant lacking Dnmt1, the enzymes responsible for CpG methylation, but not in mutants defective for either Dnmt3a or Dnmt2.

## Introduction

One of the key mechanisms of epigenetic modification of vertebrate genomes is the methylation of cytosine residues in the DNA sequence. The resulting profiles are determinant in normal developmental processes as well as in several human disorders [1], [2]. They are mitotically stable and were found in several instances inherited through the germ line [3], [4]. Methylation in the symmetric dinucleotides CpG:GpC is an important determinant of chromatin structure, playing a role in gene silencing and in the control of imprinted genes. It is maintained by the activity of DNA methyltransferase Dnmt1 on the hemimethylated meCpG:GpC structures created by replication forks [5], [6]. Although less frequent, methylation in the asymmetric non-CpG sequences CpC, CpA and CpT has been repeatedly reported, first in plant [7] and more recently in mammalian genomes [8]–[10]. The enzymes required for non CpG DNA methylation in mammals have been analyzed in ES cells where, by contrast to somatic cells, a high percentage of non CpG DNA methylation has been found. Although the Dnmt1 protein is not required for their presence, the *de novo* DNA methyltransferases Dnmt3a and/or Dnmt3b are thought to be mainly responsible for the CpA and CpT methylation [8], [11]. However, while most of the studies performed on non CpG DNA methylation in mammals, accounts for the presence of this modification at specific sites of the genome, neither the maintenance of this type of epigenetic modification over large number of generations nor the signals leading to the inheritance of this epigenetic modification have been investigated in details.

Starting from our previous observation of defined CpG and non-CpG methylation patterns induced by Cre-LoxP interaction in meiotic cells [12], we investigated the mechanisms of inheritance of these epigenetic marks. We performed a systematic analysis of one of these patterns involving both CpG and non-CpG methylations in a mutant *Rxr*α locus. Three distinct methylation profiles were evidenced at each generation, in constant ratios in all somatic tissues, were found stable over more than 15 generations. Non-CpG DNA methylation was strictly allele-specific, never affecting the wild type locus in heterozygotes. One half of the DNA molecules in this complex, at each generation and in every organ including sperm, were exclusively methylated at CpG sites, thus raising the question of the nature of the epigenetic signal transmitted with the unique copy of the sperm genome to regenerate in the next generation the constant ratio of three distinct profiles.

## Results

### Hereditary maintenance of a complex pattern of non-CpG methylation

We previously reported [12] that interaction of the Cre recombinase with a *LoxP* element in meiotic cells leads to the establishment of defined patterns of DNA methylation, extending into the neighboring sequences and involving both CpG and non-CpG sequences. To further investigate their mode of inheritance, mouse families were independently derived from parents in which such a methylated complex had been initiated at a *LoxP* element in intron 3 of the *AF1* mutant of the *Rxr*α gene. Families were separately bred for a total of about 15 generations, and DNA samples kept for analysis. A retrospective determination of the methylation status in each family at successive generations could thus be undertaken.

**Figure 1:**
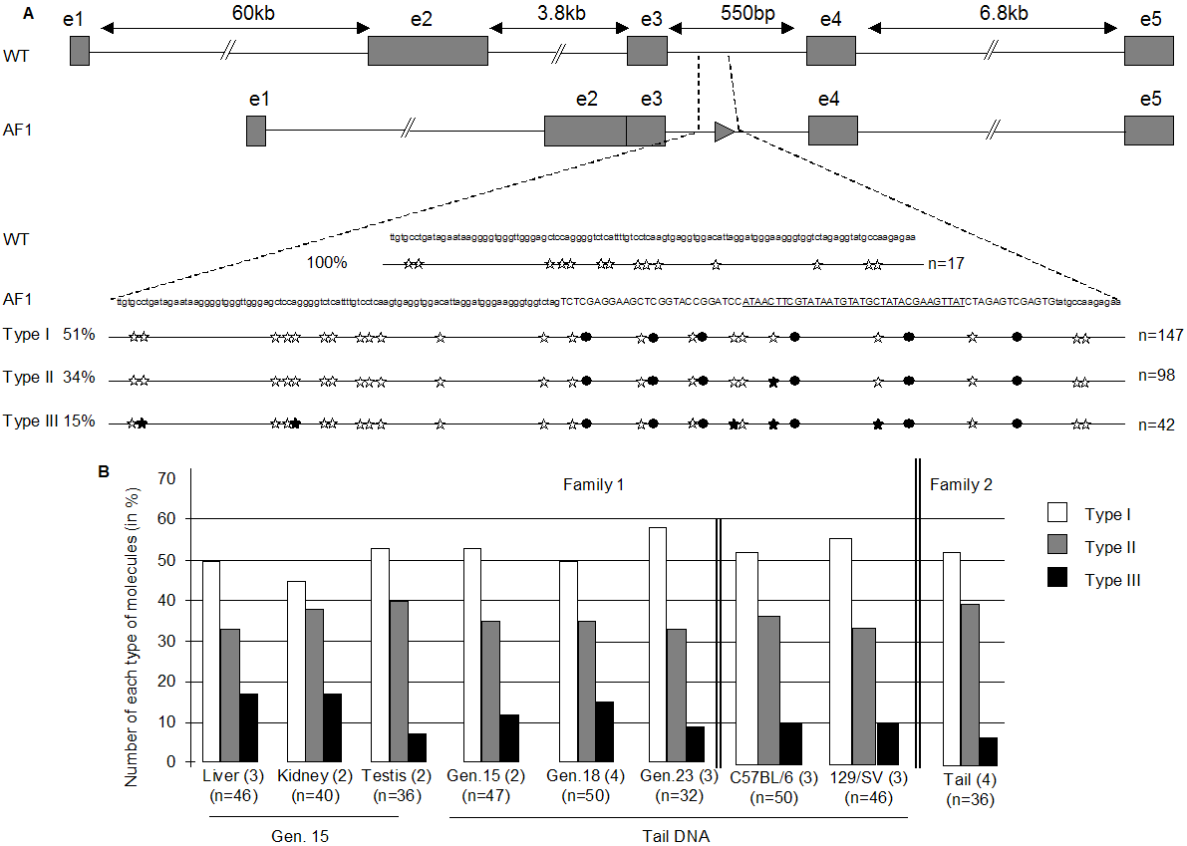
Allele-specific maintenance of a complex methylation pattern. A. Upper part: schematic representation of the *AF1* mutant and wild type *Rxr*α alleles. Boxes indicate exons. The nucleotide sequence surrounding the *LoxP* site in *AF1* (arrow head) and the methylation state of the cytosine residues are shown below. Open stars correspond to unmethyled cytosines, filled stars to methylated non-CpG and filled circles to methylated CpG sites. The three classes of methylated molecules are shown and their frequency (per cent) in a total of 287 clones sequenced after amplification. B. Distribution of the three classes of methylated molecules amplified from the *AF1* allele. Mice indicated as “Family 1” were derived from the same unmethylated *Rxr*α^AF1^ male carrying the Sycp1-Cre transgene and that indicated “Family 2”, from a distinct ancestor. Genomic DNA from organs (liver, kidney and testis) dissected from 2 to 3 independent mice and genomic tail DNA from 2 to 4 different mice of each generation were subjected independently to bisulfite treatment. Each genomic DNA were then PCR amplified separately and classified in three different classes of methylated molecules. Number of mice used in each experiment are indicated in parentheses. From left to right: Family 1 liver, kidney, testis and tail DNA after 15 generations, tail DNA after 18 and 23 generations in the B6D2 F1 genetic background; tail DNA after 8 generations of crosses into the indicated backgrounds; Family 2 tail DNA after 15 generations in B6D2 F1. n correspond to the numbers of molecules analysed.

Bisulfite analysis of a 228 bp region including the *LoxP* element evidenced a complex pattern of non-CpG methylation (Fig. 1). Three classes of methylated molecules were identified, in constant ratios in somatic organs (tail, kidney, liver) and testes at the successive generations and in distinct families. In addition to a constant pattern of CpG methylation, half of the amplified molecules were free of non-CpG methylation, one third showed one methylated CpT, and 15 per cent contained three to five methylated non-CpG cytosines, always at the same positions in the sequence (Fig 1).The modified locus was transmitted with equal high efficiencies by the male and the female parents. Families were bred in the B6D2 F1 hybrid genetic background, but the results were not dependent on this genetic set up, as the same observations were made after crosses with either C57BL/6 or 129/SV mice (Figure 1B).

The possibility of incomplete denaturation and cytosine conversion during bisulfite exposure was excluded for three independent reasons. First, artificial protection of a cytosine residue never occurs at constant sequence positions and never follows defined patterns. Secondly, non-CpG DNA methylation was specific to the mutant allele since we never evidenced such methylation in the allelic region of the wild type allele in heterozygotes (Fig 1). Finally, as a control, bisulfite sequencing analysis performed on *in vitro* methylated DNA never showed the same reproducible DNA methylation profile (data not shown).

### Transgenerational signalling of the non CpG methylation pattern

The constant frequencies of the three methylation patterns of the mutant allele were stably maintained through successive generations. We considered several mechanisms that would account for the paternal transmission of the whole complex, mediated by a single copy of the haploid genome in the compacted sperm chromatin.

**Figure 2:**
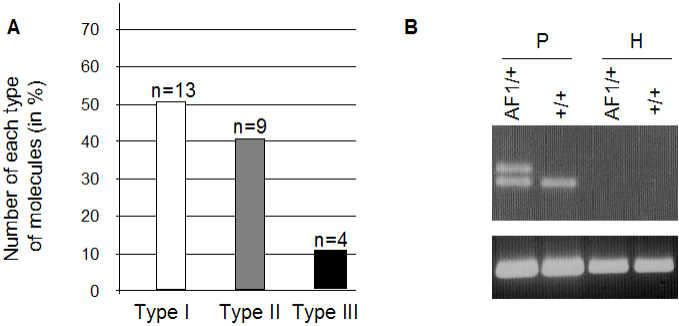
Epigenetic marks in sperm nuclei. A. Distribution in sperm DNA of the methylation profiles shown in Fig. 1. Numbers of molecules of each profile are indicated and correspond to the analysis of two independent PCR reactions where two different preparations of sperm bisulfite-treated DNA were used as template. B. The *Rxr*α locus is included in the protamine-associated fraction of the sperm chromatin. Wild type (+/+) and heterozygote (*AF1*/+) sperm chromatin preparations were partitioned into histone-enriched (H) or protamine-enriched (P) DNA fractions after *EcoR*I and *BamH*1 restriction cleavage. PCR analysis of the methylated *LoxP* region and of *Line I* elements, known to be present in the nucleosomal fraction [18], was performed on the fractions using the primers and conditions indicated in Table 1.

One possible scheme could be that the male haploid genome would carry the complete set of non-CpG methylations, part of which would be subsequently lost during somatic development. This hypothesis was disproved by bisulfite analysis of the methylation state in sperm DNA, showing a pattern in purified epididymal sperm identical to that of somatic DNA, with the same proportion of the three methylation profiles (Fig 2A). The majority of the haploid nuclei thus contains either no asymmetric methylated cytosines or only one in this interval, and still the complete picture is regenerated *de novo* at the following generation.

**Table 1:** Oligonucleotides primers for PCR amplification.

	Primers
*Rxr*α *AF1* (LoxP site)	ACAATACCTGGGCACAGCTCAC
	TGGTGGTCTTGTGCTCTCTGTG
Line	AGAGAATCTGTCTCCCAGGTCT
	TCTAGGTTCTCAGGTGTGTTGG
*Dnmt1*	CTTGTGACCAGAGGCAGAGG
	GCATCCAGACTGCCTTG
*Dnmt2*	AGAAGCCTGTGGCTTTCAGT
	CCCTACAATCGTTTATTTTCCAA
*Dnmt3a*	CATGTTGGGTCTGTTTGCTCAC
	GGGTCTTTAGCACTGCTTCCTC
	GGGTCTTTAGCACTGCTTCCTC

We then considered the somewhat less likely hypothesis that the signal leading to asymmetric methylation could be linked to the covalent modification of histones established during spermatogenesis. Inheritance via the male gamete would seem to exclude such a possibility, since histones are replaced by protamines in a generalized highly compact state of the sperm chromatin. Since, however, a minor fraction of the genome was reported to remain in the form of nucleohistones, we checked whether this might be the case of the *Rxr*α locus by fractionating histone and protamine associated DNA according to the published procedures [13], [14]. PCR analysis of the fractions showed that the *Rxr*α locus is in fact included in the nucleoprotamine fraction (Fig 2B), thus excluding histone modification as a transgenerational determinant of the modified chromatin structure.

The remaining possibility was to consider the CpG methylation pattern in the sperm genome as the determinant of non-CpG methylation. This hypothesis was strengthened by the genetic analysis of the methyltransferases involved.

### Dnmt1 activity is required to maintain the non-CpG methylation pattern

Assays of the substrate specificity of the murine DNA methyltransferases in transgenic *Drosophila*, evidenced low levels of non-CpG methylation in flies expressing either Dnmt3a or Dnmt2 [15]. The former was considered as a de novo DNA methyltransferase and a likely candidate for asymmetric methylation [8]. Dnmt2 protein has been recently shown to catalyze methylation of tRNA^Asp^ [16], but it also exhibits a limited activity of non-CpG methylation on DNA substrates.

To determine whether one of these enzymes is responsible for the maintenance of the established pattern of asymmetric methylation in *LoxP* recombined regions, we analyzed the DNA methylation status at the mutant *Rxr*α locus in mice homozygous for either a *Dnmt3a* null allele or a *Dnmt2* allele. For that purpose, *Dnmt3a* ^+/−^ or *Dnmt2* ^+/−^ heterozygotes were crossed with mice showing asymmetric methylation. Double heterozygotes were then crossed to generate the *Dnmt3a* ^−/−^ or *Dnmt2* ^−/−^ homozygotes. Bisulfite analysis (Table 2) showed that the same patterns of asymmetric methylation had been maintained at the *LoxP* allele in the *Dnmt3a* and *Dnmt2* null mutant.

**Table 2:** Heritable methylation profiles require the Dnmt1 but neither the Dnmt2 nor the Dnmt3a methyltransferase.

Methylation profile*	Mouse genotypes and DNA analysed					
	Wild type tail DNA	Wild type sperm DNA	*Dnmt 3a* ^−/−^ tail DNA	*Dnmt 2* ^−/−^ tail DNA	*Dnmt 1* ^+/−^ embryo DNA	*Dnmt 1* ^−/−^ embryo DNA
	(n = 287)	(n = 26)	(n = 23)	(n = 46)	(n = 20)	(n = 46)
	(x = 10)	(x = 3)	(x = 3)	(x = 3)	(x = 4)	(x = 3)
Type I	51±3	55±5	48±10	55±8	60±5	0
Type II	34±2	34±2	39±1	36±5	30±6	0
Type III	15±3	12±7	13±10	9±0.4	10±3	0
Other	0	0	0	0	0	100$

* CpG/non-CpG methylation profiles as shown in Fig. 1.

n and x correspond to the number of clones sequenced and the number of mice analyzed, respectively.

$ see Fig. 3.

**Figure 3:**
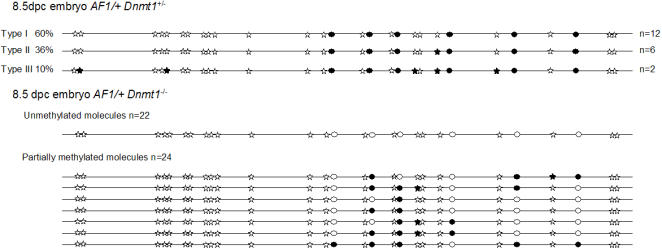
DNA methylation analysis in the modified Rxrα locus of Dnmt1^−/−^ mice. Bisulfite sequencing analysis at the *LoxP* site in 8.5 *dpc* embryos either heterozygous or homozygous for the *Dnmt1* null mutation, including representative examples of partially methylated molecules. Genomic DNA from three embryos of each genotype have been independently subjected to bisulfite treatment.

In a rather unexpected manner, the only null mutation among the methyltransferases tested that proved effective indisturbing the methylation profile of the locus was a deletion of the Dnmt1 locus. Among the four mammalian DNA methyltransferases, Dnmt1 has been considered as the main enzyme responsible for the maintenance of CpG methylation [5]. To determine whether this enzyme might be involved in the maintenance of non-CpG methylation profiles, we analyzed the status of this region in mice homozygous for a *Dnmt1* null mutation. The necessary crosses were performed to generate the methylated *Rxr*α*^AF1^*, *Dnmt1* ^−/−^ genotype. As the mutants lacking the enzyme die at mid gestation, analysis of the *Rxr*α*^AF1^*, *Dnmt1* ^−/− ^was performed on DNA extracted from embryos at 8.5 *dpc*, a developmental time at which DNA methylation patterns are well established in *Dnmt1* ^+/−^ heterozygotes as in the wild type (Fig 3). If, as expected, CpG methylation was disturbed in *Dnmt1* ^−^ homozygotes, although not completely suppressed, it was unexpectedly observed that the non-CpG profiles were abolished. The majority of the amplified molecules were either not at all or only partially methylated. Importantly, even in the latter class, none of the profiles followed the reproducible pattern of *Dnmt1* ^+^ animals.

Altogether these results suggest that asymmetric DNA methylation depends on the prior establishment of a CpG methylation profile and on its maintenance by the Dnmt1 methyltransferase.

## Discussion

DNA methylation is clearly a key feature in the establishment of local chromatin structures and epigenetic controls [1]. Still, the mechanisms leading to the establishment and maintenance of methylation patterns are largely unknown. Most of the currently available information concerns methylation in the symmetrical CpG dinucleotides. Much less is known of the somatic and germ line maintenance of the non CpG methylation pattern observed in animal and plant cells. We previously described experimental conditions under which both CpG and non-CpG methylations are established de novo in the mouse. Dependent on an initial heterologous recombination event (Cre-LoxP) in a meiotic cell, they are subsequently maintained in the absence of the recombinase. Our original observations included two loci of the mouse genome, namely the *Rxr*α and ROSA26 genes [12], and further studies were concentrated on the well-defined *Rxr*α site. As the initiation event was repeatedly reproduced from naive unmethylated mice, and families subsequently established and propagated, we were in a position to ascertain their somatic stability and heritability.

We determined that a unique CpG methylation profile is associated with a complex pattern of non-CpG methylation constituted by three distinct profiles, present in constant proportions and inherited in successive generations. Several criteria indicated a strictly controlled process. First, methylation is allele-specific, never involving the wild type allele in heterozygotes. Second, cytosines at the same positions were found methylated in animals independently generated from unmethylated parent. Third, once established, this dynamic pattern was identically reproduced in the progeny.

Substantiating a previous suggestion by Imamura et al. [17], our results suggest that non-CpG methylation depends on the prior establishment of a CpG methylation pattern, the only feature that seems to be common to all the sperm nuclei of modified males. On the other hand, based on our current understanding of the activity of Dnmt1 being primarily, if not exclusively the methylation of CpG sites [5], it seems unlikely that other cytosines could be directly recognized as substrates of the enzyme. Our results exclude Dnmt3a (Table 2), at least as the only effector, and the involvement of either Dnmt3b or 3l, as well as other chromatin-associated protein(s) able to recognize the methylated regions might also be involved, will have to be the subject of further studies. Such a model would also agree with our previous observation that, in the initial process of establishment of DNA methylation upon Cre-LoxP interaction, non-CpG methylation occurred only after the establishment of CpG methylation [12].

While CpG methylation has been associated with well defined regulatory processes such as parental imprinting and X chromosome inactivation [18] and the compact chromatine structure generated by DNA methylation is generally thought to be associated with transcriptional silencing. This is not, however, the case of the modified *Rxr*α locus. A comparative analysis of the transcripts did not evidence any interference of the epigenetic modifications with *Rxr*α transcription (data not shown). Rather than gene silencing, we favour the hypothesis that non-CpG patterns are marking genomic regions that underwent heterologous recombination, a dangerous event for a genome, as it often indicates that transposons or viral genomes are moving around. This hypothesis is coherent with the observation that Cre-LoxP recombination as well as other non homologous recombination types [12], [19] are strongly inhibited in methylated regions. It also agrees with previous observations of methylated non-CpG groups in transposons and integrated viral genomes [10], [20].

The hereditary transfer by the sperm cell of an epigenetic information, and not solely of the genomic nucleotide sequences, is in fact a more general question. While RNA molecules in the sperm chromatin may modulate gene expression in the progeny [21], paternal inheritance of epigenetic information in the form of CpG methylation patterns that in turn direct the establishment of site-specific non-CpG methylation may be but one more example in this class of non-Mendelian heredity.

## Materials and Methods

### Transgenic mice

The mouse mutants *Rxr*α*^AF1^* (*LoxP* insertion, intron 2 deletion and exon 2 partial deletion), *Dnmt1* ^+/−^ [22], *Dnmt2* ^+/−^ [16] and *Dnmt3a* ^+/−^ [23] were maintained according to the French and European regulations for the care and use of research animals. The genetic backgrounds are B6D2 F1 (Sycp1-Cre lines and progenies), C57BL/6 (*Rxr*α*^AF1^*) and 129/Sv (*Dnmt* ^+/−^ lines). Genotyping was performed by PCR analysis, with the primer sequences and amplification conditions listed in Table 1.

### Sodium bisulfite sequencing

One microgram of genomic DNA extracted from either liver, testis, kidney or tail from independent mice were restriction digested with *Bgl*II and subjected to sodium bisulfite treatment as described [24]. PCR amplification from each bisulfite-treated genomic DNA was always carried out independently using primers previously described [12]. PCR products were subcloned into the pGEM®-T Easy vector (Promega) and cycle sequenced. We sequenced 5 to 20 clones from each genomic DNA samples and performed independent sets of DNA modification, amplification, cloning, and sequencing. The sequenced obtained were classified into three categories on the basis of the methylated non-CpG content and the numbers presented in the figures correspond to the cumulative numbers obtained from each experiment.

### Fractionation of sperm chromatin

Sperm DNA was fractionated into histone-associated and protamine-associated DNA as described [13].
